# Deep Learning-Based Image Analysis for the Quantification of Tumor-Induced Angiogenesis in the 3D In Vivo Tumor Model—Establishment and Addition to Laser Speckle Contrast Imaging (LSCI)

**DOI:** 10.3390/cells11152321

**Published:** 2022-07-28

**Authors:** Paulina Mena Kuri, Eric Pion, Lina Mahl, Philipp Kainz, Siegfried Schwarz, Christoph Brochhausen, Thiha Aung, Silke Haerteis

**Affiliations:** 1 Institute for Molecular and Cellular Anatomy, University of Regensburg, 93053 Regensburg, Germany; paulina.menakuri@outlook.com (P.M.K.); ericjosh.pion@yahoo.com (E.P.); lina.mahl@stud.uni-regensburg.de (L.M.); thiha.aung@th-deg.de (T.A.); 2 KML Vision GmbH, 8020 Graz, Austria; philipp.kainz@kmlvision.com (P.K.); siegfried.schwarz@kmlvision.com (S.S.); 3 Institute of Pathology, University of Regensburg, 93053 Regensburg, Germany; christoph.brochhausen@ur.de; 4 Faculty of Applied Healthcare Science, Deggendorf Institute of Technology, 94469 Deggendorf, Germany

**Keywords:** 3D in vivo tumor model, chorioallantoic membrane (CAM), angiogenesis, tumor, laser speckle contrast imaging, image analysis software, CAM assay application, artificial intelligence, deep learning, blood circulation

## Abstract

(1) Background: angiogenesis plays an important role in the growth and metastasis of tumors. We established the CAM assay application, an image analysis software of the IKOSA platform by KML Vision, for the quantification of blood vessels with the in ovo chorioallantoic membrane (CAM) model. We added this proprietary deep learning algorithm to the already established laser speckle contrast imaging (LSCI). (2) Methods: angiosarcoma cell line tumors were grafted onto the CAM. Angiogenesis was measured at the beginning and at the end of tumor growth with both measurement methods. The CAM assay application was trained to enable the recognition of in ovo CAM vessels. Histological stains of the tissue were performed and gluconate, an anti-angiogenic substance, was applied to the tumors. (3) Results: the angiosarcoma cells formed tumors on the CAM that appeared to stay vital and proliferated. An increase in perfusion was observed using both methods. The CAM assay application was successfully established in the in ovo CAM model and anti-angiogenic effects of gluconate were observed. (4) Conclusions: the CAM assay application appears to be a useful method for the quantification of angiogenesis in the CAM model and gluconate could be a potential treatment of angiosarcomas. Both aspects should be evaluated in further research.

## 1. Introduction

### 1.1. Tumor Angiogenesis and the CAM Model

Tumor-induced angiogenesis is a central aspect in cancer research and has been the subject of a variety of studies on tumor biology. In general, tumors release pro-angiogenic factors into the tumor microenvironment that induce abnormal vessel growth [1,2,3]. Hereby, the extensive angiogenic development of tumor transplants can be attributed to the activation of host endothelial cells, which subsequently surpasses the vessel growth capacities of physiological tissue [3]. Folkman described the neovascularization of newly implanted tumor nodules as capillary sprouts that seem to rapidly extend from the host tissue toward the tumor [2]. 

Dating back as far as 1911, the chorioallantoic membrane (CAM) model has been used extensively as an angiogenesis assay for cancer-related studies [4,5,6,7,8,9]. The CAM resembles the highly vascularized membrane that is formed within fertilized chicken eggs during the embryonic development of the chicken. Apart from serving primarily as an organ for gas exchange of the chicken embryo, the CAM enables the transportation of sodium and chloride, as well as of calcium, which facilitates the mineralization of the embryonic bones [10,11]. 

Since the CAM model is a highly versatile, cost efficient and easily practicable method, it has been used for the grafting of tumor xenografts, as well as tumors derived from cell suspensions in many different applications [12,13,14,15,16,17,18,19]. The wide range of possible applications of the CAM model strongly encourages its use as a testing platform for chemotherapeutic drugs. The assessment of different aspects of tumor biology such as the effects of angiogenesis on tumor metabolism, proliferation and metastasis is also possible [20]. Furthermore, a variety of angiogenic aspects and mechanisms of the formation of tumor blood vessels have been recognized as therapeutic targets, e.g., hypoxia, as a key driver for angiogenesis [1].

The CAM model is a well-rounded xenograft model ideal to study rare and aggressive tumors such as angiosarcomas. Angiosarcomas represent a tumor entity known to be especially aggressive with regards to local reoccurrence and metastasis. They are classified as a group of soft tissue sarcomas that are derived from lymphatic or vascular endothelial cells and are associated with a very poor prognosis [21,22]. Different versions of the CAM model have previously been used to successfully graft and culture angiosarcoma cell lines and test potential treatment agents [23,24,25]. 

### 1.2. Previous Measurement Methods of Angiogenesis in the CAM Model

The measurement of angiogenesis is of upmost importance when testing chemotherapeutics, particularly those with potential anti-angiogenic effects [26]. Angiogenesis measurement methods applied to the different variants of the CAM model include the manual counting of vessels in defined areas (so-called vasculogenic index) [27], the semiautomated image quantification with an ImageJ plug in in addition to the vasculogenic index [28] and other software applications such as AngioTool [29]. In addition, histological analyses [30], injection of fluorescent [31] and non-fluorescent markers [32], MRI or PET imaging, optical Doppler tomography and many other methods [10] can be used. 

Additionally, laser-based methods present a frequent method for the measurement of angiogenesis, which is why we established an angiogenesis measurement protocol based on laser speckle contrast imaging (LSCI) for the CAM model in a previous study [33]. 

### 1.3. Novel Measurement Method for the Quantification of Angiogenesis in the CAM Model

Our goal in this study is to enable the use of a deep learning-based angiogenesis quantification software for the assessment of the angiogenesis of angiosarcomas in the in ovo CAM model, which to the best of our knowledge has not been performed so far. Therefore, a recently developed and user-friendly measurement method that is provided by image analysis software could pose a more sufficient and easier-to-operate tool for the assessment of angiosarcoma-induced angiogenesis. This method is part of the proprietary IKOSA platform (KML Vision GmbH, Graz, Austria) which is a commercially available software-as-a-service solution for digital image data management and analysis. It currently offers 12 ready-to-use applications and can be accessed from a simple web browser. Several of them focus on the automated quantification of vascular structures in recorded microscopic images, one of which is the so-called CAM assay application. It was initially created in 2020 by the company in collaboration with the Medical University of Vienna and the Medical University of Graz with the aim of replacing previously performed analyses by manually counting the vessels in the ex ovo CAM model. We wanted to use this application and expand its use to the analysis of angiosarcoma-induced angiogenesis in the in ovo CAM model, which to our knowledge has not been done so far.

### 1.4. LSCI as an Established Second Independent Angiogenesis Measurement Method

To test the sufficiency of the application, we additionally utilized LSCI as a second independent angiogenesis measurement method technique, which solves the problem of quantifying angiogenesis by directly measuring the blood flow through the computation of the contrast fluctuations of a so-called speckle pattern [34]. Hereby, LSCI is a non-invasive, laser-based method that has already been used to investigate microcirculatory blood perfusion of different tissues in a variety of studies, including studies that used the CAM model [15,35,36,37,38]. The underlying principle of LSCI comprises the analysis of the contrast in a so-called speckle pattern that is caused by the backscattering of laser light from the movement of erythrocytes in blood vessels. This provides information on the amount of movement that is relative to the amount of blood flow within the vessels.

### 1.5. Testing of Anti-Angiogenic Agents on Angiosarcomas in the CAM Model

Gluconate represents an anti-angiogenic substance and has previously been described to deprive pancreatic cancer cells of citrate, which significantly reduced the growth, metastasis and angiogenesis in vivo [39]. Previous data describes gluconate as a competitively binding specific antagonist for the pmCiC transporter (plasma membrane citrate carrier). PmCiC, which is highly expressed in tumor cells, is responsible for providing citrate to the mitochondrial citrate cycle of the tumor cells [40,41,42]. 

The aim of our study was to establish the CAM assay application of the IKOSA platform as a novel method for the analysis of tumor-induced angiogenesis in the in ovo CAM model. We optimized and used the CAM assay application, as well as LSCI, independently to evaluate the effects of an anti-angiogenic substance on the cell metabolism of angiosarcoma cell line tumors on the CAM. Therefore, we trained the software with microscopic images of CAMs that were grafted with angiosarcoma cells and analyzed the vasculature of the tumors using the application. To evaluate the sufficiency of the novel methodology, we added the measurement results of our established LSCI protocol and described a combined protocol for a more versatile analysis of angiogenesis [15]. Furthermore, we treated grafted angiosarcoma tumors in the CAM model with gluconate and were able to demonstrate its anti-angiogenic effects using both independent methods. 

## 2. Materials and Methods

### 2.1. CAM Model

The CAM model was performed according to previous descriptions of our established protocol [33,43,44]. Fertilized chicken eggs (n = 75) were rinsed with water, brushed and disinfected with 70% ethanol. Afterward, they were immediately placed on a rotation device inside a ProCon egg incubator (Grumbach, Asslar, Germany) at a constant temperature of 37.8 °C, a pCO_2_ of 5% and with the humidity calibrated to 63%. On the fourth day of incubation, two windows were cut into the eggshell. For the first window, the air entrapment of the egg was localized with a gooseneck lamp (usually at the pointy end of the egg) and marked before cutting the eggshell with sharp pincers and a small pair of scissors. This first window, with an approximate size of 1 cm × 1 cm, enabled an equilibrium of pressure inside the egg and was permanently closed with Leukosilk^®^ (BSN medical, Hamburg, Germany). The second window was cut into the longitudinal part of the egg and covered with a removable strip of Leukosilk^®^. It had an approximate size of 1 cm × 1 cm and was used for tumor grafting and angiogenesis measurements. Afterward, the eggs were placed on glass trays containing autoclaved sand in the non-rotating compartment of the egg incubator.

At the beginning of the angiogenesis measurements, the second window was enlarged to an adequate measurement window of approximately 1.5 cm × 2 cm if considered necessary. On the nineth day of incubation, the CAMs were grafted with angiosarcoma tumor cell suspensions before the eggs were placed back on the glass trays filled with autoclaved sand in the egg incubator for two days to facilitate tumor growth. 

On the 11th day of incubation, the first angiogenesis measurement utilizing LSCI and the CAM assay application took place. On the 16th day of incubation, the second and last angiogenesis measurement was performed using both methods. 

### 2.2. Engraftment of AS-M Cells on the CAM

The angiosarcoma (AS) cell line AS-M [45] was purchased from DSMZ (German collection of microorganisms and cell cultures GmbH, Braunschweig, Germany). The cells were cultivated in endothelial cell growth medium (passage numbers p27–32). For grafting, the cells were counted with a Neubauer counting chamber and aliquots of 2 × 10^6^ cells were mixed with 30 µL Matrigel. Afterward, the Matrigel cell suspensions were hardened at a temperature of 37 °C for 3–4 min before transferring them onto a prepared CAM using a spatula. The preparation of the CAM involved the roughening of a superficial vessel of medium diameter in the center of the CAM with a clean cotton swab until visible micro bleeding was observed. This ensured the adherence of the Matrigel cell suspensions to the CAM. Two days later the formation of a coherent tumor mass was observed.

### 2.3. Training and Establishment of the CAM Assay Application for the Application in the in ovo CAM Model

The CAM assay application consists of a prediction model and a postprocessing step and is provided on the IKOSA platform as an analysis module. The prediction model is based on a state-of-the-art convolutional neural network (CNN) algorithm [46] that resembles an end-to-end image segmentation model for the recognition of blood vessels from raw image data. The network architecture is inspired by a U-net structure [47], which uses an encoder path to extract features from the image before expanding again in the decoder path to predict an image of the same size as the input. Each pixel in this image represents a score ranging between 0 and 1, representing the membership of the pixel to the class of “blood vessel”. By postprocessing this score map using a threshold at 0.5, a binary segmentation mask of the vessels is obtained, where morphological parameters can be extracted. Four parameters are computed and provided as readouts: total vessel area, total vessel length, mean vessel thickness and number of branching points. 

However, version 1.0.0 of the CAM assay application was originally developed for the ex ovo setting of the CAM model and did not perform well on in ovo images; therefore, a retraining of the model was performed. In a novel set of 15 microscopic images of the in ovo CAM with engrafted tumors, the vascular structures were labeled using freehand and polygon annotation tools provided by IKOSA. The training and validation dataset consisted of 10 and 5 images, respectively, and was used to improve and evaluate the vessel recognition performance. Training input data was augmented by applying random shifts in color, space and spatial transformations (flipping, rotations) to artificially extend the variance and make the model more robust. The existing CNN model (v1.0.0) was carefully fine-tuned using a binary cross-entropy loss function on the new data and converged in about nine hours on a graphics processing unit. Training stopped early without overfitting at a point, where no significant improvement could be observed anymore; see Supplementary Materials for details (Figure S1). The final model resulted in the following performance metrics on the validation dataset: Dice score of 0.83, pixel-wise precision of 0.79 and pixel-wise recall of 0.88. The postprocessing routine remained unchanged. 

The improved application version (CAM Assay v3.0.0) was made available on the platform in parallel to the original CAM Assay v1.0.0 and the photographs were analyzed regarding the vascularization of the CAM. 

### 2.4. First Independent Angiogenesis Measurement Technique: The CAM Assay Application of the IKOSA Platform

The first angiogenesis measurement with the CAM assay application, last used on 11 April 2022, was performed on the 11th day of incubation. Therefore, microscopic images of the CAM were taken on the same day using a Leica M205A microscope. Afterward, these microscopic images of the CAM were uploaded onto the IKOSA platform using a web browser and analyzed by the improved CAM assay application v3.0.0. When a new image is submitted for analysis, the platform schedules the execution of the application, collects the results, and presents the quantitative and qualitative output data to the user. This takes only a few seconds and the procedure is fully automated. The second angiogenesis measurement of the CAM with the CAM assay application was performed on the 16th day of incubation in the exact same way as the first. The CAM assay application can be accessed as a free trial by signing up to the IKOSA platform at https://app.ikosa.ai.

### 2.5. Second Independent Angiogenesis Measurement Technique: Laser Speckle Contrast Imaging (LSCI) 

The first angiogenesis measurement of the CAM with LSCI took place on the 11th day of incubation. We used the commercially available PeriCAM perfusion speckle imager (PSI) system high resolution (HR) model (PERIMED, Järfälla, Sweden) for the LSCI-based methodology. The integrated PIMSoft software uses arbitrary perfusion units [PU] to depict the average movement in the blood vessels detected in the individual recorded images. The measurement was performed according to the protocol established by Pion et al. [33]. This included recording each egg until 10 consecutive images were taken during which the embryo remained still and averaging the data to receive the representative perfusion of each egg on the day of measurement. The real-time graph provided by PIMSoft is based on the single perfusion values of the recorded images. The PeriCAM PSI system uses a near-infrared laser (NIR) (785 nm, 80 mW) to scan the CAM, which is supposed to be located at a distance of 10 cm from the laser head. An additional visible laser (650 nm, 3 mW) marks the area scanned by the NIR laser and helps with the positioning of the egg. The measurement frame was set to depict an area of 1 cm × 1.1 cm on the CAM with the tumor in the center. Images were taken at a frame rate of one image per second. 

The second angiogenesis measurement with LSCI was performed according to the same protocol on the 16th day of incubation. To ensure an accurate detection of blood vessels a filter of 800–3000 PU was applied to all perfusion measurements [15]. 

### 2.6. Histological Analysis

At the end of the experiment, the tumors were removed from the CAM and prepared for fixation and staining. The tumors were put in paraformaldehyde (PFA) at −2 °C under constant movement for approximately 4 days until fixation was observed. After removing the PFA, the tumors were immersed in 4% PBS (phosphate buffered saline, pH 7.2) for 30 min at −2 °C under constant movement. This procedure was repeated three times. Consequently, the tumors were dehydrated and fixed in paraffine. Tissue samples of a thickness of 6 µm were cut using a microtome and embedded and stained with hematoxylin and eosin (H&E staining). 

### 2.7. Treatment with Sodium Gluconate

For the study of the anti-angiogenic and cytotoxic effects of sodium gluconate on the AS-M cells, we engrafted CAMs (n = 75) with a suspension of 2 × 10^6^ AS-M cells and treated them with 30 µL of sodium gluconate (150 µM, 0.9% solution) after engraftment, which was applied evenly on and around the tumor lesion. Treatment of the tumor cells took place daily from the 11th to the 16th day of incubation (ctrl, n = 36; glc, n = 39).

The control group was treated with 30 µL ddH_2_O in the same manner. On the 16th day of incubation, sections of the CAMs of both groups containing the tumors, each with an equidistant CAM-brim around the lesion, were removed from the CAM to examine the previously inaccessible bottom of the CAM, as well as the blood vessels that had been covered by the tumor.

### 2.8. Statistical Methods

GraphPad Prism 8 (Dotmatics, Boston, MA, USA) was used for the statistical analysis and the creation of graphs. The mean ± standard deviation was calculated. The significance of the acquired data was assessed with the appropriate version of one-way ANOVA and a Tukey’s multiple comparisons post hoc test. The significance level was set at *p* < 0.05. 

## 3. Results

### 3.1. Growth of Angiosarcoma Cell Line Tumors on the CAM 

**Figure 1:**
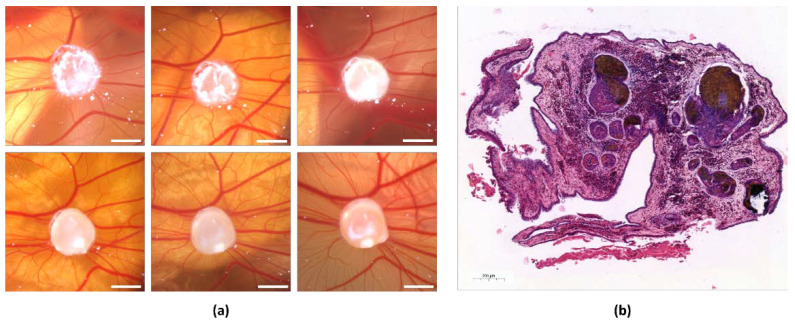
Growth of AS-M cell line tumors on the CAM and H&E staining. (**a**) Series of photographs of angiosarcoma cell line tumors on the CAM from the top left to the bottom right showcasing each day from the 11th until the 16th day of incubation. The white bar indicates 2 mm. (**b**) H&E staining of an AS-M cell line tumor after removal from the CAM after one week.

The tumor-induced angiogenesis of the AS-M cell line tumors on the CAM was macroscopically observed over the course of 5 days by taking photos with a Leica microscope. We observed an increase in the density of the blood vessels that grew toward the tumor radially. Furthermore, the diameter of the vessels visibly increased and new capillaries were formed (Figure 1a). By performing two measurements on two different days, an accurate analysis of the newly formed vasculature can be assured. The apparent growth of the AS-M cell line tumors was displayed through adequate size development and induction of angiogenesis on the CAM. Tumor growth partially took place underneath the CAM. The CAM surrounding most tumors appeared vital in color and did not dry out throughout the experiment (Figure 1a). The attachment of tumors to the CAM and growth upon implantation was successful as evident in hematoxylin and eosin (H&E) stained tumor tissue samples after the removal of tumors from the CAM (Figure 1b).

### 3.2. Establishment and Improvement of the CAM Assay Application with Deep Learning

**Figure 2:**
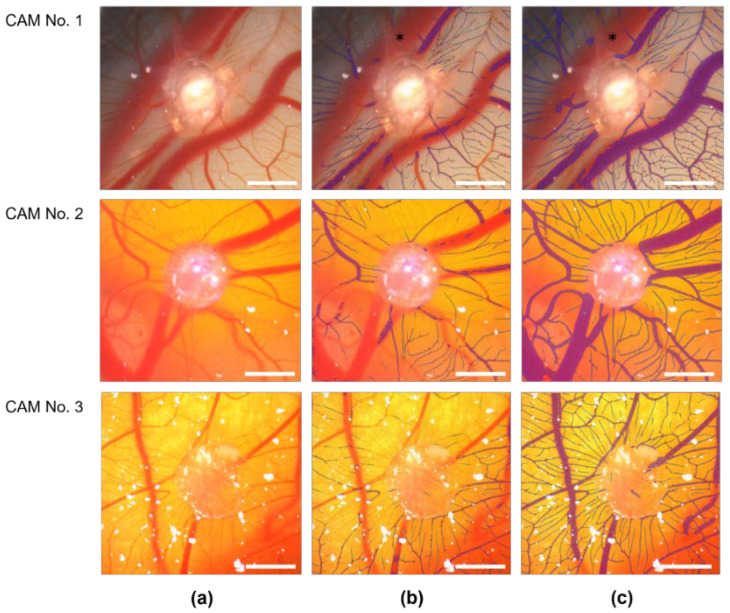
Visualization of the development of the CAM assay application with three different CAMs and engrafted AS-M cell line tumors (the white bar indicates 2 mm). (**a**) Original microscopic images of the CAMs with the grafted AS-M cell line tumor in the center. (**b**) Analysis of the microscopic images of CAMs with engrafted tumor cells with the CAM assay application v1.0.0 or (**c**) with the CAM assay application v3.0.0.

For the analysis of the microscopic photographs of the CAM taken with the Leica microscope regarding four different angiogenic parameters (total vessel area, total vessel length, mean vessel thickness and number of branching points), we utilized the CAM assay application of the IKOSA platform. To assess the performance of the improved version of the algorithm (v3.0.0) we compared its results to data of the original version of the application (v1.0.0). The blood vessels in the microscopic images (Figure 2a) that were recognized by the CAM assay application are marked in purple in the resulting image (Figure 2b,c). The first software version (v1.0.0) was insufficient in adequately detecting both relatively thick vessels, as well as thinner vessels (Figure 2b). Due to the updates of the new software version, (v3.0.0) those issues were mostly resolved and the thin vessels now made up a significant part of the angiogenesis measurement. Subsequently, an improvement of the sensitivity for the recognition of blood vessels on all levels was obvious (Figure 2). Furthermore, vessels underneath the CAM, marked by * in Figure 2b,c, may appear blurrier than the superficial ones. To ensure that the deeper vessels were not part of the final measurements, training data contained representative examples of such vessels.

**Figure 3:**
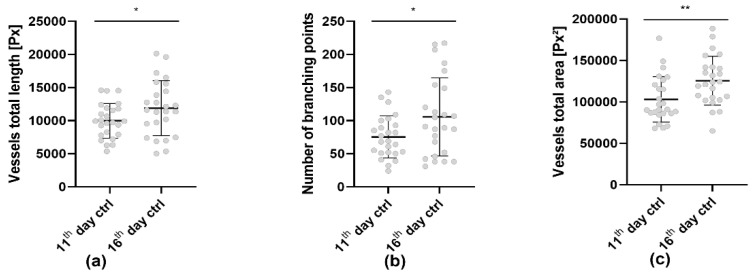
Increase in perfusion detected by the CAM assay application v3.0.0 of 25 CAMs grafted with AS-M cell line tumors (n = 25). (**a**) Total vessel length detected by the CAM assay application. (**b**) Branching points detected by the CAM assay application. (**c**) Total area of all vessels detected by the CAM assay application. All three data sets were analyzed using one-way ANOVA with Tukey´s multiple comparisons test (* *p* < 0.05, ** *p* < 0.01).

For the evaluation of the angiogenesis induced by the engrafted tumors, we focused on the parameters of total vessel length (Figure 3a), number of branching points (Figure 3b) and total vessel area (Figure 3c) as they appeared to be the most suitable for the analysis of angiogenesis of the CAM. Using the new CAM assay application v3.0.0 we observed a significant increase in perfusion from the 11th to the 16th day of embryonic development, indicated by all three parameters (Figure 3). The total vessel length (Figure 3a) increased from 1.00 × 10^4^ ± 0.26 × 10^4^ px to 1.19 × 10^4^ ± 0.41 × 10^4^ px. Meanwhile the number of branching points (Figure 3b) increased from 77.40 ± 31.78 to 105.70 ± 59.08. The total vessel area increased from 10.31 × 10^4^ ± 2.76 × 10^4^ px^2^ on the 11th day to 12.56 × 10^4^ ± 2.95 × 10^4^ px^2^ on the 16th day (Figure 3c). 

### 3.3. Analysis of the Tumor Induced Angiogenesis via LSCI

**Figure 4:**
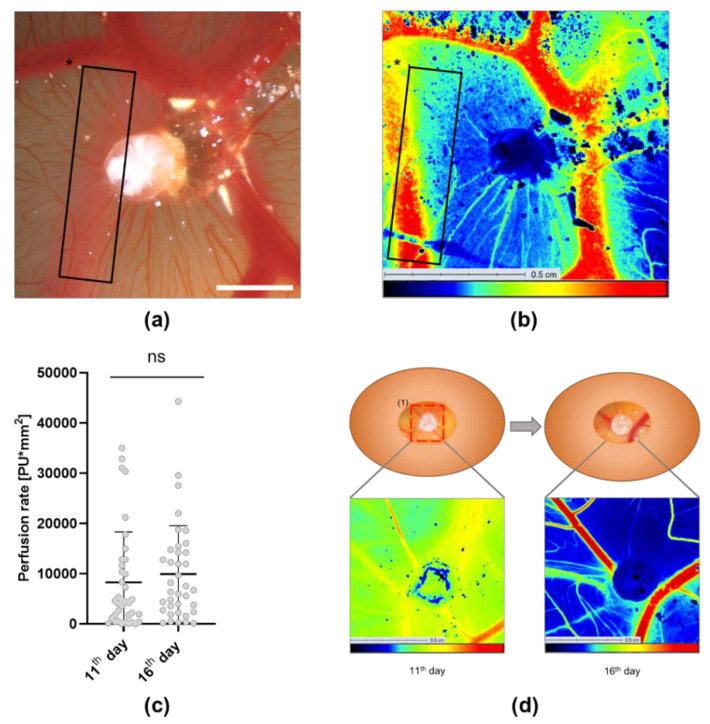
Analysis and illustration of the LSCI-based angiogenesis measurements of CAMs grafted with AS-M cell line tumors (the white bar indicates 2 mm). (**a**) The vessel marked with a * in the microscopic image is blurry because of its location underneath the surface of the CAM but is (**b**) partially recognized by LSCI. (**c**) Scatterplot diagram showcasing the mean blood flow of the CAM calculated by multiplying the perfusion rate (PU) and the perfused area (mm^2^) shows an increase from the 11th to 16th day of incubation after applying a perfusion filter of 800–3000 (PU) to the LSCI measurements. The data was evaluated using one-way ANOVA with Tukey´s multiple comparisons tests (n = 36). The significance level was set at *p* < 0.05. (**d**) Illustration of the measurement protocol for the LSCI measurements of the CAM models.

**Figure 5:**
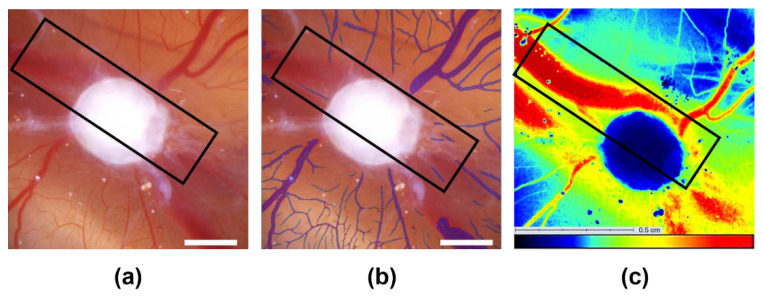
Location-dependent recognition of blood vessels by the CAM Assay application and LSCI (the white bar indicates 2 mm). (**a**) Original microscopic image. (**b**) Analysis using the CAM assay application v3.0.0. The vessels marked in purple were recognized by the application. The deep vessel which is marked by a black rectangle is not recognized by the CAM assay application. (**c**) Perfusion image of the same CAM with an AS-M cell line tumor in the center. The same vessel marked by the black rectangle is clearly included in the LSCI-based measurement.

The angiogenic development of the CAM and tumor-induced blood vessels was measured with LSCI on the 11th and 16th day of incubation through a window in the eggshell. The eggs were positioned in accordance with the microscopic images (Figure 4a,b). The mean blood flow on the 11th day was calculated by multiplying the measured perfusion area with the measured perfusion value after applying a perfusion filter [15] and resulted in a value of 0.84 × 10^4^ ± 1.02 × 10^4^ PUmm^2^. On the 16th day, the mean blood flow had increased to 1.01 × 10^4^ ± 0.97 × 10^4^ PUmm^2^ (Figure 4c,d). We observed that vessels underneath the CAM were recognized by LSCI but remained unconsidered by both software versions provided by IKOSA (Figure 4a,b and Figure 5). 

### 3.4. Assessment of Angiogenesis after Treatment with Gluconate Using LSCI and the CAM Assay Application

**Figure 6:**
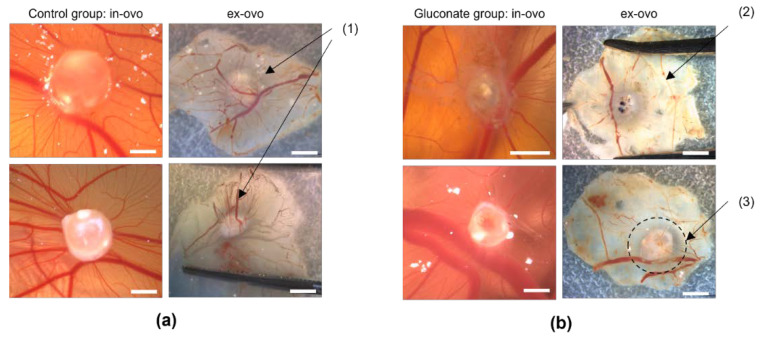
Effect of sodium gluconate on angiogenesis of AS-M cell line tumors on the CAM. (**a**) Microscopic images as well as photographs of untreated tumors grafted onto the CAM. The arrow marks the dense and radiant vascularization of the tumors on the bottom side after removal from the CAM. The white bar indicates 2 mm. (1) indicates that the CAM surrounding the tumor is densely vascularized. (**b**) Microscopic images as well as photographs of tumors that were treated with sodium gluconate after engraftment on the CAM. The arrows mark the absence of blood vessels surrounding the tumor on the bottom side after removal from the CAM, which corresponds to the area where sodium gluconate was applied to (3). (2) indicates rudiments of thin blood vessels that previously supplied the tumor. The vessel rudiments appear to be atrophic.

We treated 39 CAMs with sodium gluconate and 36 CAMs in the control group with ddH_2_O. We then compared the results of the control group (Figure 6a) and the gluconate group (Figure 6b). Each CAM was observed in ovo and ex ovo after excision to evaluate the blood supply on the bottom of the tumors as well. The bottom side of control CAMs (Figure 6a) showed a dense and radiant vascularization of the tumors with embryonic blood vessels growing toward and inserting into the tumor implant (marked as (1) in Figure 6a), which is typical for tumor transplants on the CAM. In comparison, the vascularization as seen in in ovo images of the treated group looked similar to the controls (Figure 6b). However, the ex ovo images showed structures that appeared to be rudiments of vessels as well as a lack of thin vessels inserting into and surrounding the tumor (marked by (2) in Figure 6b) corresponding to the section of the CAM where gluconate was applied ((3) in Figure 6b). 

**Figure 7:**
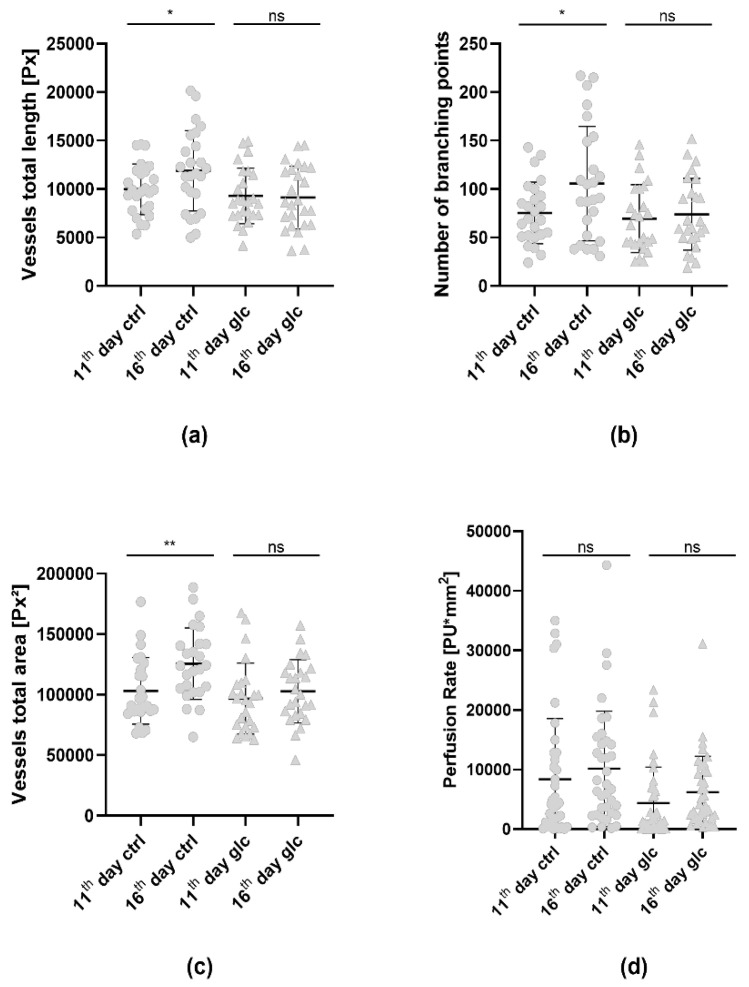
Graphs depicting the angiogenic development of the AS-M cell line tumors on the CAM measured with LSCI and the CAM assay application. Mean and standard deviation are included. (**a**) Length of all vessels, (**b**) number of branching points and (**c**) area of all vessels as measured by the CAM assay application version 3.0.0 from the IKOSA platform (ctrl, n = 25; glc, n = 25). (**d**) Perfusion status of the treated and the control group measured by LSCI. We used the appropriate version of one-way ANOVA with Tukey´s multiple comparisons test for all datasets (significant * *p* < 0.05, ** *p* < 0.01). (ctrl, n = 36; glc, n = 39).

Furthermore, the angiogenic development was measured on the 11th and 16th days of incubation using the CAM assay application and LSCI. The mean total vessel length quantified by the CAM assay application (Figure 7a) of the control group significantly increased from 1.00 × 10^4^ ± 0.26 × 10^4^ px to 1.19 × 10^4^ ± 0.41 × 10^4^ px. However, the mean total vessel length of CAMs treated with gluconate showed a nonsignificant decrease from 0.93 × 10^4^ ± 0.29 × 10^4^ px to 0.91 × 10^4^ ± 0.32 × 10^4^ px from the 11th to the 16th day. The mean number of branching points quantified by the CAM assay application of the control group increased from 77.40 ± 31.78 on the 11th day to 105.70 ± 59.08 on the 16th day (Figure 7b). Meanwhile, the mean number of branching points of the CAMs treated with gluconate showed a nonsignificant increase from 69.52 ± 35.02 on the 11th day to 73.92 ± 37.00 on the 16th day. Finally, the area of all vessels (Figure 7c) provided by the CAM assay application showed a significant increase of 10.31 × 10^4^ ± 2.76 × 10^4^ px^2^ on the 11th day to 12.56 × 10^4^ ± 2.95 × 10^4^ px^2^ on the 16th day within the control group. The gluconate group showed a nonsignificant increase from 9.66 × 10^4^ ± 2.93 × 10^4^ px^2^ to 10.27 × 10^4^ ± 2.62 × 10^4^ px^2^.

On the other hand, the data acquired with LSCI showed a nonsignificant increase in the mean perfusion of the control CAMs on the 11th day from 0.84 × 10^4^ ± 1.02 × 10^4^ PUmm^2^ to 1.01 × 10^4^ ± 0.97 × 10^4^ PUmm^2^ on the 16th day (Figure 7d). LSCI measurements of the gluconate treated CAMs also showed a nonsignificant increase from 0.43 × 10^4^ ± 0.61 × 10^4^ PUmm^2^ to 0.62 × 10^4^ ± 0.61 × 10^4^ PUmm^2^. 

## 4. Discussion

It was our aim to establish a novel quantification method to measure tumor-induced angiogenesis in the in ovo CAM model. Therefore, we grafted angiosarcomas onto the CAM and measured the angiogenesis at the beginning and at the end of the experiment with the CAM assay application of the IKOSA platform. By applying both the CAM assay application and our already established LSCI protocol we were able to show how two independent methods can be used simultaneously to achieve a more comprehensive understanding of angiogenesis of angiosarcomas on the CAM. Another additional aspect that we added to our study design was the application of gluconate, an antiangiogenic substance that led to a significant inhibition of the tumor-induced angiogenesis. Previous studies that involved investigations of the tumor-induced angiogenesis specifically of angiosarcomas cultivated in the CAM model have made use of so-called angiogenic or vasulogenic indexes. These indexes describe the percentage of blood vessels in a defined area as measured by fluorescent microscopy [48]. Furthermore, stereomicroscopic images combined with histological staining have been used for the evaluation of proliferation of angiosarcomas grown on the CAM [49]. However, both methods cannot be performed non-invasively since they include staining and snap freezing of the tissue, which is time-consuming and expensive. Other applications made use of non-invasive angiogenesis quantification software such as the Metamorph software [50]. However, this software was only applied to angiosarcomas grown on the ex ovo CAM model and it requires configuration of the detectable vessel parameters, making it less user-friendly and more prone to inadequate data acquisition. Apart from variations from the in ovo CAM model regarding the exposed surface area and the background, the mortality and risk of infection, as well as the material expense, are significantly higher in the ex ovo model, which makes this application less desirable [26]. Other possibilities for the quantification of angiogenesis on the CAM include immunoblots [51] and histology [52], as well as fluorescence microscopy, which enables the detection of angiogenic distinctions between healthy and tumor tissue [53]. However, as these applications make use of surrogate parameters that are prone to errors, they may not adequately represent the level of angiogenesis.

The emergence of digital image analysis software programs that are characterized by an automated analysis based on artificial intelligence represents a rapidly growing field that affects almost all aspects of health care with an emphasis on subjects such as radiology or pathology [30,31,32,33]. These programs have the potential of becoming highly useful and robust tools for the classification and categorization of data acquired by assessing the blood vessel networks in microscopic images of the CAM. 

The convolutional neural network (CNN) is currently one of the most used artificial neural networks and also represents the basis of the CAM assay application of the IKOSA platform. The CNN, as a specific set of deep learning networks, was first introduced by Lecun et al. [54] and is specifically well-rounded for the analysis of images [55,56,57]. In their novel and highly technical publication, Lok et al. optimized the imaging of microvasculature with the help of a CNN and used the CAM model to prove the application in an in vivo setting was possible [58]. However, the study remains technical and focuses on the imaging modalities of the CNN. Unlike our presented work, it does not include biological factors such as tumor-induced angiogenesis or the effects of anti-angiogenic substances. Other recent and more technical publications include the use of the CAM model for the application of ultrasound localization microscopy, which utilizes a CNN to visualize microvessels [59,60]. Li et al. talk about the difficulty of obtaining the large in vivo training datasets required to train previously used cross-correlation-based localization methods combatted by the improved learning scheme of CNNs, though only using ex ovo CAM assay training data. Regardless, deep learning-based ultrasound localization techniques still lack any further developments aside from the in ovo application such as tumor grafting, larger numbers of CAM models or the application of other external substances. With this work, we take the technical developments of CNNs one step farther toward preclinical testing of potential chemotherapeutics with the in ovo CAM model. 

Our goal was to establish the CAM assay application as a CNN-based image analysis method for the quantification of angiogenesis and to continuously adapt it for the application to the in ovo model with a sufficient level of vessel recognition performance with in ovo images. Hereby, we wanted to improve the algorithm and add it to our established angiogenesis measurement protocol on the basis of LSCI. Both applications could pose a valuable combination for the assessment of angiogenesis and could also be used for the examination of angiosarcoma cell line grafted CAMs. 

Regarding all parameters offered by the CAM assay application, it is up for debate which of these parameters can be seen as the most valid indicator for different types and subtypes of angiogenesis. 

The mean vessel thickness, which showed no significant change during the microcirculatory development of both the control and the gluconate tumors (Figure S2), describes an average that expresses both the growth of existing vessels and neovascularization. Since LSCI (Figure 4 and Figure 7d) describes the additive total perfusion rather than an average value of angiogenesis, we decided that the mean vessel thickness was not a suitable comparison to LSCI for our experimental model. 

Furthermore, the deeper blood vessel marked with * in Figure 4a,b remains undetected using both the original and the new CAM assay application even after the software has been trained to recognize vessels of similar size. In conclusion, the CAM assay application was not sufficient in detecting blood vessels underneath the CAM. LSCI, on the other hand, guarantees the recognition of blood vessels underneath the CAM as long as they are located close to the membrane and display a high perfusion status. This can be attributed to the fact that LSCI has to reach a penetration depth of approximately 300 µm when used for the measurement of dermal blood flow [61,62,63]. Therefore, the CAM assay application does not fully display the angiogenic development of deeper vessels and LSCI could be used as an additional tool here. It may also be questionable how much the CAM tumors depend on blood supply from superficial CAM vessels in contrast to vessels that enter the tumor from underneath the CAM. 

Yet, after training the CAM assay application algorithm with a variety of in ovo CAM images with and without tumors, the CAM assay application v3.0.0 was able to detect blood vessels of relatively small diameter in a sufficient manner, i.e., between 3 and 220 px. This enabled the differentiation of angiogenesis of angiosarcomas that were treated with sodium gluconate and the controls. Nevertheless, the fact that LSCI can be used for the actual measurement of blood flow instead of only evaluating the blood vessels based on their visual appearance cannot be neglected [35,64]. Rather, the methods can be used complementarily to generate deeper insights.

For the examination of the sensitivity, as well as the most suitable application form, we treated engrafted tumors and the surrounding CAM with sodium gluconate, a substance previously described to impede tumor cell metabolism and angiogenesis [40,41,42]. Following treatment with gluconate, three parameters (total vessel length, number of branching points and total vessel area) indicated a decrease in angiogenesis. Since the CAM assay application v3.0.0 mainly recognizes blood vessels of smaller diameter, it can be assumed that those are mostly affected by the treatment with sodium gluconate. While results of LSCI measurements primarily correspond to vessels with wide diameter (Figure 7d), the results generated by the CAM assay application are more strongly affected by changes in the configuration of small vessels than the LSCI-acquired results (Figure 7a–c). We observed an increase in perfusion measured with LSCI for both the controls and the treated group (Figure 7d), which may be caused by the gluconate treatment that mainly affects thinner vessels that are excluded by the application of a perfusion filter [15]. 

Furthermore, when microscopically analyzing thicker and rather superficial vessels with the CAM assay application, they seem to not have been affected by the gluconate application as described in the experimental model. Nevertheless, larger datasets will be needed to fully assess the anti-angiogenic effects of gluconate on the vasculature in the CAM model using the CAM assay application. We also recommend further studies regarding the anti-angiogenic effects of gluconate on angiosarcomas. 

## 5. Conclusions

In conclusion, the CAM assay application v3.0.0 is a highly valuable tool for the assessment of angiogenesis in the in ovo CAM model. The proposed model could be used as a drug-screening application for a variety of cancer types and therefore contribute to the increasingly pursued practice of personalized medicine. In addition, gluconate could be a potential treatment option for angiosarcomas because of its anti-angiogenetic effects. We recommend further studies regarding these aspects.

## Data Availability

Data is contained within the article or supplementary material. The data presented in this study are available in Deep Learning-Based Image Analysis for the Quantification of Tumor-Induced Angiogenesis in the 3D In Vivo Tumor Model—Establishment and Addition to Laser Speckle Contrast Imaging (LSCI).

## Supplementary Materials

The following supporting information can be downloaded at https://www.mdpi.com/article/10.3390/cells11152321/s1: Figure S1: Training and validation loss of the CNN model retraining for the in ovo applications as a function of training time; Figure S2: Graph depicting the mean vessel calculated by the CAM assay application version 3.0.0.
