# Supplementary material for: Deep Learning-Based Image Analysis for the Quantification of Tumor-Induced Angiogenesis in the 3D In Vivo Tumor Model—Establishment and Addition to Laser Speckle Contrast Imaging (LSCI)
Source: Cells. 2022 Jul 28;11(15):2321. doi: 10.3390/cells11152321 (PMC9367525; doi:10.3390/cells11152321)
Supplement: Supplementary file 1 [file cells-11-02321-s001.zip › cells-1803879-supplementary.pdf]

Supplementary Materials

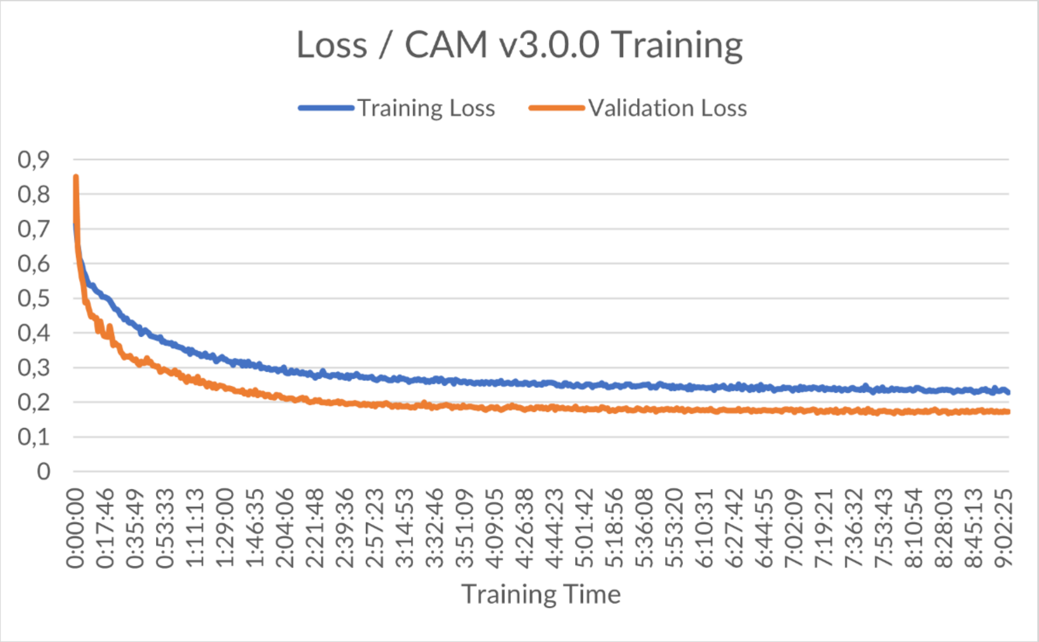

Figure S1. Training and validation loss of the CNN model retraining for the in-ovo applications as a function of training time. New annotated microscopy image data of in-ovo CAMs was presented using color space and spatial (flipping, rotating) augmentation techniques during supervised training to increase the variance. After each training step, which lasted on average about 17.5 minutes, the model's generalization performance was evaluated on the validation data. Both training and validation loss continued to decrease over the training time, indicating a good convergence of the model. Training stopped automatically after about 9 hours, when no improvement on the validation loss could be observed anymore to avoid overfitting.

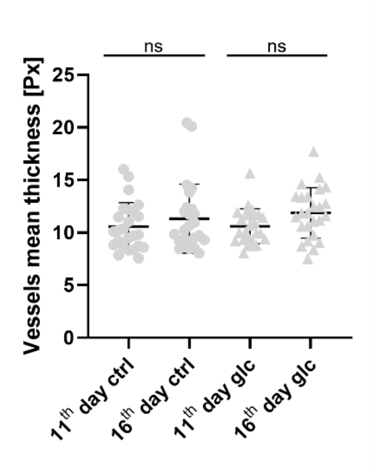

Figure S2. Graph depicting the mean vessel thickness calculated by the CAM Assay application version 3.0.0**.** The application was used on AS-M cell line tumor grafts on the CAM. The scatterplot compares the control group and the gluconate group (ctrl, n = 25; glc, n = 25). We used the appropriate version of one-way ANOVA with Tukey´s multiple comparisons test for the data set (the significance level was set at $p < 0.05$). Neither the control group, with a thickness of 10.57 ± 2.27 px on the 11th day and 11.33 ± 3.27 px on the 16th day, nor the gluconate group, with an increase from 10.59 ± 1.66 px to 11.86 ± 2.40 px, showed significant changes.
